# Application of bi-directional long-short-term memory network in cognitive age prediction based on EEG signals

**DOI:** 10.1038/s41598-023-47606-7

**Published:** 2023-11-18

**Authors:** Shi-Bing Wong, Yu Tsao, Wen-Hsin Tsai, Tzong-Shi Wang, Hsin-Chi Wu, Syu-Siang Wang

**Affiliations:** 1 Department of Pediatrics, Taipei Tzu Chi Hospital, Buddhist Tzu Chi Medical Foundation, New Taipei City, Taiwan; 2 School of Medicine, Tzu Chi University, Hualien, Taiwan; 3 Research Center for Information Technology Innovation, Academia Sinica, Taipei, Taiwan; 4 Department of Psychiatry, Taipei Tzu Chi Hospital, Buddhist Tzu Chi Medical Foundation, New Taipei City, Taiwan; 5 Department of Physical Medicine and Rehabilitation, Taipei Tzu Chi Hospital, Buddhist Tzu Chi Medical Foundation, New Taipei City, Taiwan; 6 Department of Electrical Engineering, Yuan Ze University, Taoyuan, Taiwan

**Keywords:** Neurophysiology, Biomedical engineering

## Abstract

Electroencephalography (EEG) measures changes in neuronal activity and can reveal significant changes from infancy to adulthood concomitant with brain maturation, making it a potential physiological marker of brain maturation and cognition. To investigate a promising deep learning tool for EEG classification, we applied the bidirectional long short-term memory (BLSTM) algorithm to analyze EEG data from the pediatric EEG laboratory of Taipei Tzu Chi Hospital. The trained BLSTM model was 86% accurate when identifying EEGs from young children (8 months–6 years) and adolescents (12–20 years). However, there was only a modest classification accuracy (69.3%) when categorizing EEG samples into three age groups (8 months–6 years, 6–12 years, and 12–20 years). For EEG samples from patients with intellectual disability, the prediction accuracy of the trained BLSTM model was 46.4%, which was significantly lower than its accuracy for EEGs from neurotypical patients, indicating that the individual’s intelligence plays a major role in the age prediction. This study confirmed that scalp EEG can reflect brain maturation and the BLSTM algorithm is a feasible deep learning tool for the identification of cognitive age. The trained model can potentially be applied to clinical services as a supportive measurement of neurodevelopmental status.

## Introduction

From infancy to adulthood, humans experience significant behavioral, emotional, and cognitive development that is closely correlated with brain maturation. The development of the brain, including neuronal genesis, migration, organization, and myelination, originates in the third week of gestation and continues into the second decade of life^1^. However, the disruption of normal brain maturation induces various neurodevelopmental disabilities, such as intellectual disability (ID) and autism spectrum disorder (ASD)^2^. ID, which affects 1–3% of the population, describes a group of people with subaverage intellectual and adaptive functions^3^. Therefore, people with ID have cognitive ages incomparable with their true chronological ages. For example, despite their chronological age, people with moderate ID can mostly function only at a cognitive age of 6–8 years old. This suggests that their brain maturation has been impaired because of various genetic or neurological etiologies^3^. Although adult brain magnetic resonance imaging (MRI) can help identify intelligence and cognitive age^4,5^, brain MRI findings in children with ID are highly heterogeneous and not recommended as a routine examination^6^.

Electroencephalography (EEG) is a neurophysiological test that measures changes in neuronal activity associated with current flow in the brain^7^. Concomitant with brain growth, myelination, expanding connectivity, and maturation, scalp EEG from infancy to adulthood reveals significant changes^8^. For example, awake basal cortical rhythm, consisting of mixed-frequency activity in the neonatal stage, is replaced by rhythmic theta waves with frequencies from 3–4 Hz at 3 months old increasing to 6–7 Hz at 1 year old, and gradually transforming into a posterior-dominated alpha rhythm after 8–10 years old^7–9^. The impairment of cortical rhythm maturation with age is correlated with neurodevelopmental abnormalities, which are evidenced by immature EEG and low developmental scores in small-gestational-age infants^10^ and an increased risk of ASD in infants with tuberous sclerosis complex^11^. These observations potentially make EEG a physiological marker of brain maturation and cognition; however, there is currently no objective interpretation of EEG changes from infancy to adolescence.

Several investigators attempted to use EEG signals to predict brain age. By extracting 102 features from six EEG channels in routine polysomnography, Sun et al. proposed the “brain age index” to indicate the difference between brain age and the individual’s chronological age. A high brain age index, which indicates excessive brain aging, was found in patients with significant neurological and psychiatric diseases^12^, and it also predicted a reduced life expectancy^13^. Dimitriadis et al. introduced a brain age classifier using an extreme learning machine, a type of feedforward artificial neural network. This classifier effectively differentiated between two age groups: young adults (aged 18–37 years) and middle-aged adults (aged 46–60 years) with accuracy rates of 97% for EEG data recorded during eye-open conditions and 87% for EEG data acquired during eye-closed resting states^14^. However, there have been relatively few studies examining the prediction of brain age in children and adolescents using EEG-related methods. By analyzing resting-state EEG during the waking period, machine learning models, including random forest and relevance vector machines, achieved greater than 94% accuracy when classifying individuals into childhood (5–7 years) and adolescence (16–18 years) groups^15^.

Besides CNN approaches, recurrent neural networks (RNNs) and long short-term memory (LSTM) models were also successfully employed in the task of age estimation. Kaushik et al. utilized LSTM to develop brain-computer interfaces and predict age and gender using EEG signals^16^. In their evaluation, they achieved impressive prediction accuracies of 93.7% for age and 97.5% for gender. Another study by Jusseaume et al. compared multiple LSTM-based models for age prediction across subjects ranging from 2 to 88 years old, and a significant finding was that the bi-directional LSTM (BLSTM) model outperformed the standard LSTM in terms of age estimation accuracy^17^. Their summary also indicated that BLSTM exhibited superior prediction capabilities compared to the previously discussed CNN models. Moreover, RNNs and LSTM models have found applications in various domains, such as emotion recognition and epileptiform spike detection from EEG samples, with accuracies surpassing 80%^18,19^. Furthermore, LSTM is widely used for stress detection^20,21^, Parkinson’s disease detection^22^, motor imagery classification^23^, and epileptic seizure recognition^24^ based on diverse and non-linear EEG input signals^25^. However, it is important to note that the EEG data used in these studies lacked detailed neuropsychiatric profiles, which introduces an important bias in brain-age prediction.

In addition to traditional deep learning models, recent advancements in deep learning have explored the use of Transformer frameworks originally developed for natural language processing in the age estimation task^26^. He et al. proposed a global–local transformer approach, where features were extracted from localized pixel points and large regions of an MRI image, and then fed into a transformer model to generate brain age predictions^27^. On the other hand, Cai et al. utilized structural MRI and diffusion tensor imaging images in a graph transformer model to achieve age estimation^28^. While the successful application of transformers in estimating brain age from MRI images has been demonstrated, their effectiveness in predicting age from EEG data remains unknown. Further research is needed to explore the potential of Transformers in EEG-based age estimation.

The primary objective of this study is to establish an EEG-based cognitive age prediction method in children and adolescents using deep learning models, specifically BLSTM and transformer models. For this purpose, we collected EEG data from neurotypical individuals at Taipei Tzu Chi Hospital, and their neurological development was assessed through interviews conducted by experienced physicians. With this dataset, our aim was to develop a cognitive age classifier capable of distinguishing EEGs from young children, older children, and adolescents. Additionally, we obtained EEG data from patients with neurodevelopmental disorders who underwent structured psychometric evaluations. We hypothesized that the EEGs of these patients would be classified into age groups younger than their chronological age due to their impaired cognitive abilities. Ultimately, our goal is to develop an assessment tool that can identify brain maturation patterns from childhood to adolescence, while also facilitating early detection of potential brain disorders.

## Methods

### Participants

We performed BLSTM and Transformer training and testing in three steps which is described in the result section. We collected 375 EEG samples of patients, with age ranging from 8 months to 20 years, exhibiting no significant neurological or psychiatric disorders, and 58 EEG samples of patients, with age ranging from 8 months to 20 years, exhibiting neurodevelopmental disorders including cerebral palsy (CP), ID, or ASD, from the pediatric EEG laboratory of Taipei Tzu Chi Hospital. In this study, we have categorized EEG samples into three distinct age groups for the purpose of model training and testing. These groups encompass young children (8 months–6 years, corresponding to ages before elementary school), older children (6–12 years, representing elementary school students), and adolescents (12–20 years, encompassing high school students). The rationale behind this stratification is rooted in the varying cognitive abilities exhibited across these age cohorts, thus facilitating potential clinical applicability. The study was conducted in accordance with the Declaration of Helsinki and was approved by the Local Ethics Committee of Taipei Tzu Chi General Hospital (07-XD-095). Written informed consent was waived because of the retrospective analysis of the EEG samples.

### EEG acquisition

EEG recordings were acquired using an EEG machine (Neurofax EEG-1200; Nihon Kohden, Tokyo, Japan) with Ag/AgCl electrodes at a sampling rate of 200 Hz. A total of 19 electrodes were placed by a trained research technologist according to the International 10–20 system. All electrodes were referenced to the ground electrode in the Fpz position during the recording. After visual inspection of artifact rejection, a 10-s EEG recording during the eye-closed waking state was retrieved for further analysis.

### Data processing

We first applied a filter to process each EEG channel and provide a temporal sequence that contained only low-frequency (0–25 Hz) components of the original input. These low-frequency EEG channels were then placed at the predictor input while obtaining the predefined human stage at the system output.

### BLSTM method

The stage predictor was composed of a BLSTM deep learning model^29,30^. Specifically, BLSTM applies two LSTMs to extract the EEG features. The first LSTM was used to process the input sequence from the beginning to the end, while the other was applied to handle the same input but reverse its time flow. Each LSTM involves a recursive process, where, at each time point, the input EEG vector is weighted by weighting functions to output the extracted signal feature. This feature, which contains statistical information, is then passed to the next time point to generate a new estimation and EEG feature. In addition, a three-gate operation is implemented in a LSTM for controlling the information flow, giving the BLSTM algorithm the advantage of isolating the temporal characteristics of different brain activities. Subsequently, the BLSTM passes the concatenating LSTM outcomes to a feedforward layer. Following this, the BLSTM is able to model sequential dependencies between input and output in both directions of the sequence^31,32^.

We applied four cascaded BLSTMs to extract the EEG features to be used for the following two feedforward layers. Depicted long-term and localized EEG signal structures were considered jointly to shrink unimportant components and thus preserve the essential and representative features for the following cluster network^33,34^. The model structure used in the study was i) 256, 128, 64, and 32 cells used sequentially to construct hidden LSTM layers, and ii) a feedforward model comprising two hidden layers in the order of 32 and 16 nodes. As a result, the number of trainable parameters amounts to 531,939, which is closely resembling the BLSTM model employed in Kaushik et al.’s investigation^16^.

### Transformer model

The key element of a Transformer model is a multi-head self-attention block. In this study, the Transformer consists of four attention blocks followed by a flatten operator and a feed-forward model with two hidden layers (in the order of 32 and 16 nodes). In addition, a layer normalization operation is applied between attention blocks. Meanwhile, (head size, element size) are (256, 8), (128, 8), (64, 8) and (32, 8) in order of applying attention blocks^26^. The parameter count stands at 328,387. Remarkably, this model structure has demonstrated superior performance in our preliminary testing.

### Statistical analyses

Statistical analyses were performed using SPSS version 19.0 (IBM Corporation, Armonk, NY, USA). Descriptive data are presented as mean ± SD. Chi-squared tests and independent t-tests were used to compare factors such as age, sex, EEG indications, and classification accuracies between different deep learning models, EEG samples of different chronological ages and from EEG samples of neurotypical and neurodevelopmental individuals. Multivariate stepwise linear regression models were used to explore the neurodevelopmental factors associated with classification accuracy, including ID, ASD, and CP. Two-sided *P* values < 0.05 were considered to be statistically significant.

### Ethical approval and informed consent

The study was conducted in accordance with the Declaration of Helsinki and was approved by the Local Ethics Committee of Taipei Tzu Chi General Hospital (07-XD-095). Written informed consent was waived because of the retrospective analysis of the EEG samples.

## Results

### Classification accuracy of an individuals’ chronological age

**Figure 1:**
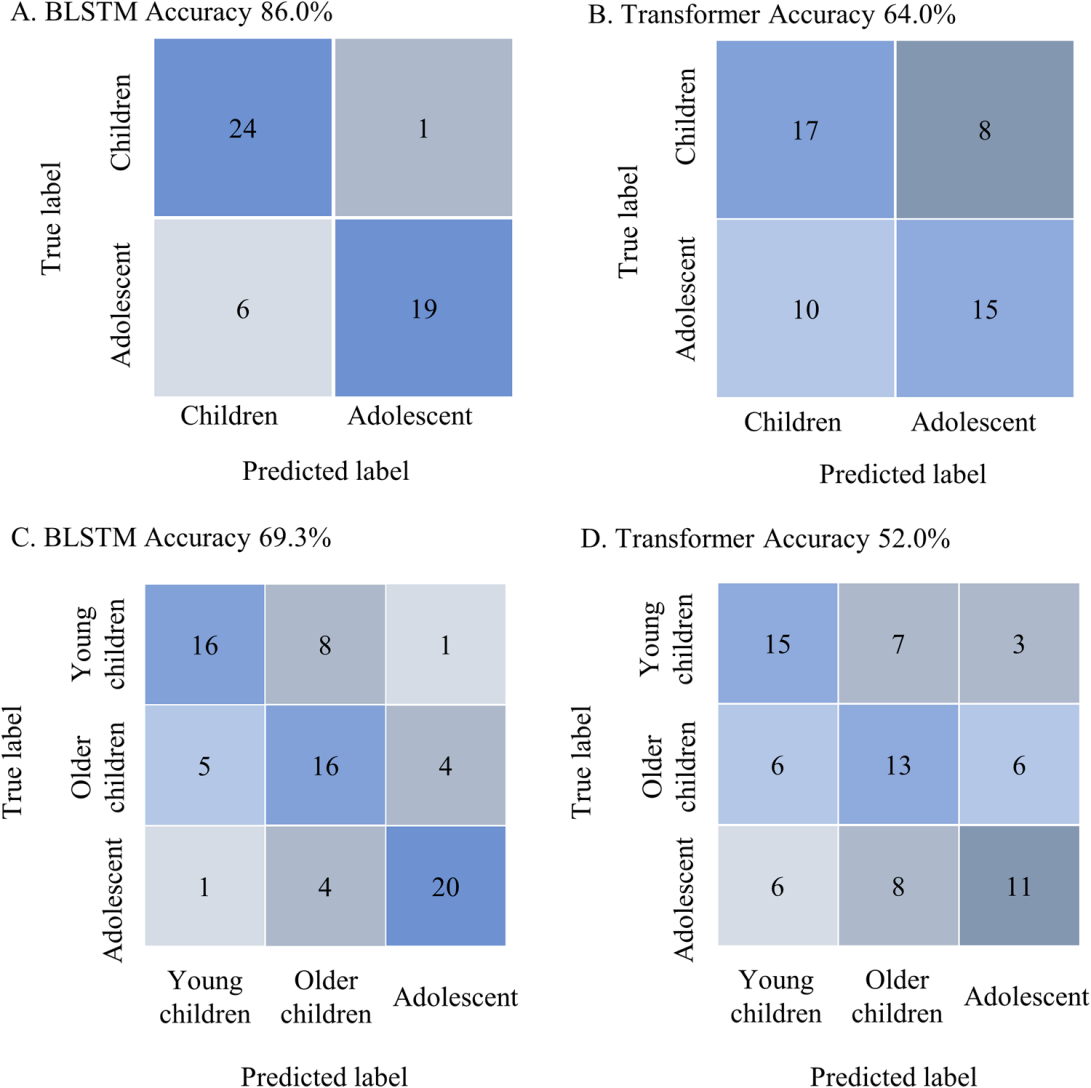
Confusion Matrices of classification accuracies of Bidirectional long short-term memory (BLSTM) and Transformer model for patients’ chronological ages using (**A**, **B**) two groups: children vs. adolescents, and (**C**, **D**) three groups: young children, older children, and adolescents.

To establish a cognitive age classifier, we initiated the study by collecting EEG data from two distinct age groups: young children (8 months–6 years) and adolescents (12–20 years). We anticipated achieving a high classification accuracy due to the notable differences in baseline EEG rhythms between these two groups. We retrospectively collected 250 EEG samples of young children (8 months–6 years) and adolescents (12–20 years). We assigned 200 EEGs to a training group and 50 EEGs to a testing group (4:1). In both groups, half the patients were young children and half were adolescents. The age, sex, and EEG indications showed no differences between the two groups (Table 1). After training, the BLSTM algorithm successfully identified 24 of 25 EEG samples of young children (96% accuracy) and 19 of 25 EEG samples of adolescents (78% accuracy) in the testing group. The overall accuracy of the BLSTM when classifying individuals’ as young children or adolescents was 86% (Fig. 1A). In contrast, the Transformer algorithm identified 17 of 25 EEG samples of young children (68% accuracy, Fig. 1B) and 15 of 25 EEG samples of adolescents (60% accuracy, Fig. 1B). The overall accuracy of the Transformer was 64% which is significantly lower than BLSTM model (*P* = 0.011, chi-square test).

**Table 1:** Patient training and testing group demographic data of EEG samples of young children (8 months–6 years) and adolescents (12–20 years).

	Training (n = 200)	Testing (n = 50)	*P*-value
Baseline characteristics			
Age (SD)	9.3 (6.0)	9.4 (6.3)	0.936^a^
Sex (M/F)	113/87	27/23	0.750^b^
Indication for EEG (%)			0.108^b^
Seizure	69 (34.5)	20 (40.0)	
Headache	49 (24.5)	6 (12.0)	
Tics	20 (10.0)	4 (8.0)	
Syncope	13 (6.5)	1 (2.0)	
ADHD	21 (10.5)	11 (22.0)	
Others	28 (14.0)	8 (16.0)	

IQR, interquartile range; ADHD, attention deficit hyperactivity disorder; EEG, electroencephalography.

^a^Independent t-test.

^b^Chi-squared test.

In the subsequent phase, we incorporated an additional 125 EEG samples from patients aged 6 to 11 years. This augmentation expanded our EEG dataset to include a total of 375 patients spanning the age range of 8 months to 20 years. Our objective was to develop a cognitive age classifier capable of accurately categorizing EEG samples into three distinct groups: young children (0–5 years), older children (6–11 years), and adolescents (12–20 years). The age, sex, and indications for EEG of three different age groups were shown in Table 2. The indications for EEG varied significantly among the three age groups. Specifically, a larger proportion of adolescents underwent EEG due to headaches, whereas children were more frequently referred for EEG studies related to ADHD or tic disorders (Table 2). We assigned 300 EEGs to the training group and 75 EEGs to the testing group (4:1). The age, sex, and indications of the EEGs showed no differences between the training and testing groups (Table 3). The trained BLSTM algorithm showed 64% classification accuracy for younger children (16/25), 64% accuracy for older children (16/25), and 80% accuracy for adolescents (20/25; Fig. 1C). The overall prediction accuracy was 69.3% (52/75; Fig. 1C). In contrast, the Transformer model exhibited an overall classification accuracy of 52% (39/75, Fig. 1D), which was notably lower than the accuracy achieved by the BLSTM model (*P* = 0.030, chi-square test). Therefore, based on the same EEG dataset, we concluded that the BLSTM model is more suitable for predicting cognitive age compared to the Transformer model. Additionally, we conducted an in-depth analysis of patients who received accurate and inaccurate predictions of their chronological age by BLSTM. Our examination of factors such as age, sex, and indication of the EEGs in these two patient groups revealed no significant differences (Table 4).

**Table 2:** Demographic data of patients in younger children, older children and adolescents groups.

	Young Children (n = 125)	Older Children (n = 125)	Adolescent (n = 125)	*P*-value
Baseline characters				
Age (SD)	3.6 (1.5)	8.8 (1.8)	15.0 (2.2)	< 0.001^a^
Sex (M/F)	78/47	79/46	62/63	0.050^b^
Indication for EEG (%)				< 0.001^b^
Seizure	53 (42.4)	13 (10.4)	36 (28.8)	
Headache	9 (7.2)	45 (36.0)	46 (36.8)	
Tics	17 (13.6)	28 (22.4)	7 (5.6)	
Syncope	0 (0)	6 (4.8)	14 (11.2)	
ADHD	22 (17.6)	26 (20.8)	10 (8.0)	
Others	24 (19.2)	7 (5.6)	12 (9.6)	

IQR, interquartile range; ADHD, attention deficit hyperactivity disorder. EEG, electroencephalography.

^a^ANOVA.

^b^Chi-square test.

**Table 3:** Patient training and testing group demographic data of EEG samples of younger children (0–5 years), older children (6–11 years), and adolescents (12–20 years).

	Training (n = 300)	Testing (n = 75)	*P*-value
Baseline characteristics			
Age (SD)	9.2 (5.0)	9.1 (5.3)	0.908^a^
Sex (M/F)	176/124	43/32	0.834^b^
Diagnosis (%)			0.196^b^
Seizure	81 (27.0)	21 (28.0)	
Headache	85 (28.3)	15 (20.0)	
Tics	41 (13.7)	11 (14.7)	
Syncope	18 (6.0)	2 (2.7)	
ADHD	40 (13.3)	18 (24.0)	
Others	35 (11.7)	8 (10.7)	

IQR, interquartile range; ADHD, attention deficit hyperactivity disorder.

^a^Independent t-test.

^b^Chi-squared test.

**Table 4:** Characteristics of patients with correctly and erroneously predicted chronological ages.

	Correct prediction (n = 52)	Erroneous prediction (n = 23)	*P*-value
Baseline characteristics			
Age (SD)	9.7 (5.6)	7.9 (4.2)	0.174^a^
Sex (M/F)	27/25	16/7	0.154^b^
Indication for EEG (%)			0.848^b^
Seizure	13 (25.0)	8 (34.8)	
Headache	12 (23.0)	3 (13.0)	
Tics	8 (15.4)	3 (13.0)	
Syncope	1 (1.9)	1 (4.3)	
ADHD	13 (25.0)	5 (21.7)	
Others	5 (9.6)	3 (13.0)	

IQR, interquartile range; ADHD, attention deficit hyperactivity disorder. EEG, electroencephalography.

^a^Independent t-test.

^b^Chi-squared test.

### Accuracy of BLSTM for classifying brain maturation in patients with neurodevelopmental disorders

**Figure 2:**
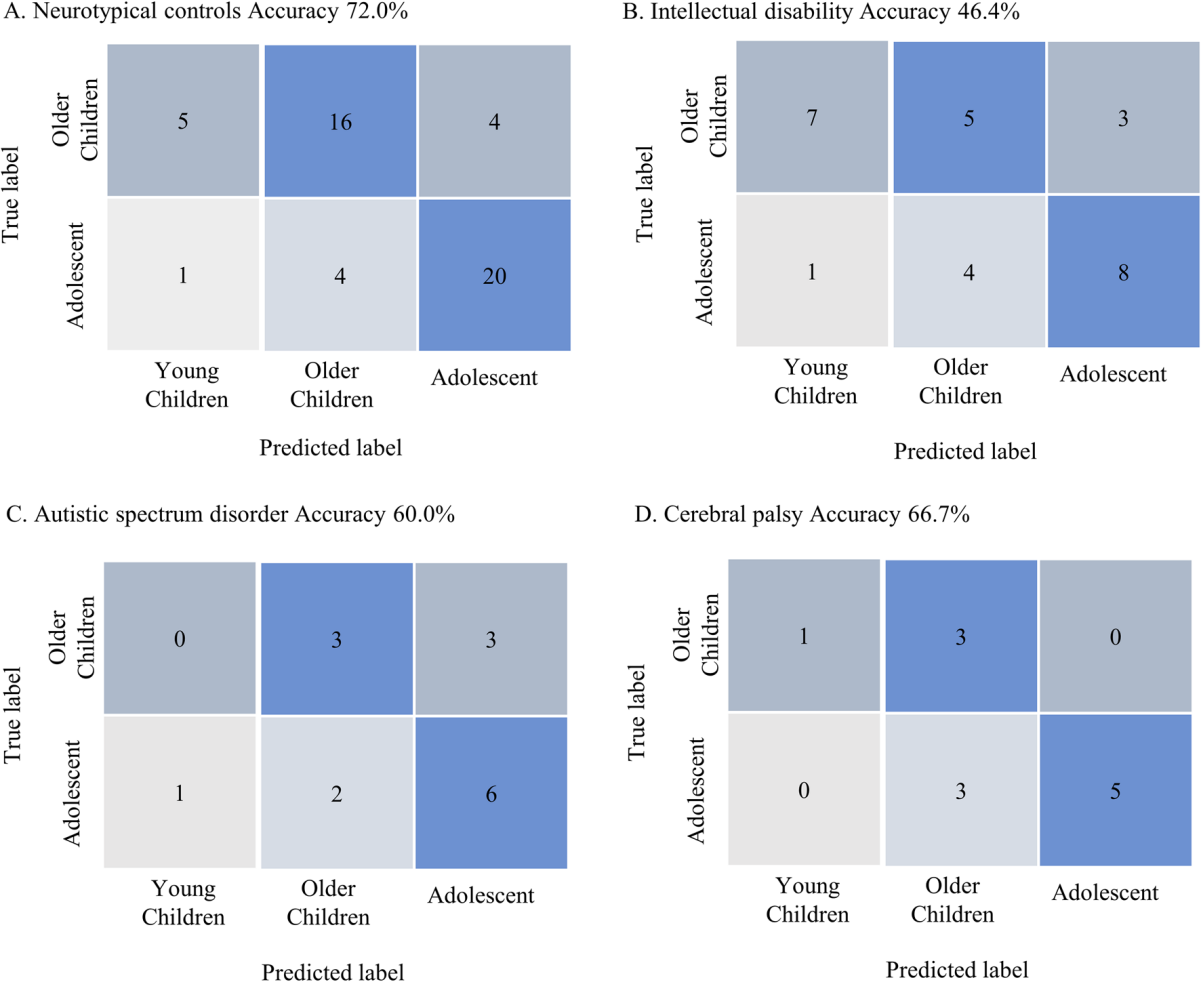
Confusion Matrices classification accuracy of Bidirectional long short-term memory for the chronological ages of patients with different neurological disorders including neurotypical subjects (**A**), intellectual disability (**B**), autistic spectrum disorder (**C**), and cerebral palsy (**D**).

In this experiment, we applied the BLSTM algorithm trained to classify individuals’ ages into three groups to identify the brain age of EEG samples from patients with neurodevelopmental disorders. We presumed that these EEG samples would reflect the impaired brain development of these patients and lead to incorrect age classifications. For example, the EEG of an 8-year-old patient with ID might be identified as a 5-year-old, corresponds more to their cognitive age. We collected 37 EEG samples from patients older than 6-years old with neurodevelopmental disorders and compared their classification accuracy with that of neurotypical individuals (n = 50). Among these patients, 28 had been diagnosed with ID, 15 with ASD, and 12 with CP. For neurotypical individuals, the classification accuracy was 72% (36/50; Fig. 2A). In contrast, the classification accuracies for patients with ID, ASD, and CP were 46.4%, 60%, and 66.7%, respectively (Fig. 2). Compared with neurotypical participants, the BLSTM algorithm significantly decreased the classification accuracy of EEGs from patients with ID (*P* = 0.025; chi-squared test). This result was in line with our hypothesis that brain age classified by the BLSTM algorithm is related to cognitive age but not chronological age. In this EEG dataset, some patients had two or more diagnoses (Supplementary Fig. 1). Therefore, we applied a multivariate logistic regression analysis to evaluate factors, including ID, ASD, and CP, for the correct prediction of brain maturation using BLSTM. After multivariate regression analysis, the factor ID still revealed a significant effect on the age prediction, with an odds ratio of 0.231 (95% CI 0.074–0.724, *P* = 0.012; Table 5). In the final step, we collected 21 EEG samples from 0 to 5-year-old patients (the younger children group) with ID. We presumed that these patients would have lower mental ages compared to their chronological ages, and it would be likelier that the BLSTM algorithm would identify brain age in the younger children group. As expected, the classification accuracy of this EEG dataset was 76.2%, which was slightly better than the result for young neurotypical individuals which was 64%. This finding further consolidated our hypothesis that brain age identified by BLSTM can better reflect a patients’ cognitive age but not their chronological age.

**Table 5:** Multivariate stepwise logistic regression analysis of the association between the classification accuracy of bidirectional long short-term memory and patients’ neuropsychiatric symptoms.

	Model 1		Model 2		Model 3	
	OR (95% CI)	*P*-value	Adjusted OR (95% CI)	*P*-value	Adjusted OR (95% CI)	*P*-value
Intellectual disability	0.322 (0.126, 0.824)	0.018*	0.259 (0.087, 0.768)	0.015*	0.231 (0.074, 0.724)	0.012*
Autistic spectrum disorder	–	–	1.762 (0.456, 6.812)	0.411	1.976 (0.489, 7.993)	0.339
Cerebral palsy	–	–	–	–	1.715 (0.414, 7.100)	0.457

Model 1: unadjusted.

Model 2: adjusted for autistic spectrum disorder (category).

Model 3: adjusted for autistic spectrum disorder (category) and cerebral palsy (category).

*P < 0.05.

## Discussion

In this study, we used EEGs to create an evaluation tool for assessing cognitive age. We trained a BLSTM algorithm with EEGs from neurotypical children and adolescents, achieving 86% accuracy when separating the two groups. However, accuracy dropped to 69.3% when separating EEGs into younger and older children. This may be due to individual variation in cognitive ability, which is distributed even among neurotypical children. We then used EEGs from patients with neurodevelopmental disorders to confirm our hypothesis that LSTM can determine cognitive ability from EEGs, and found that ID was the predominant factor affecting classification accuracy. Further research with full IQ profiles is necessary to develop a practical tool for clinical use.

To our knowledge, there is only one study conducted by Vandenbosch et al. that utilized EEG samples to predict brain maturation in the pediatric age group^15^. In their study, they employed Random Forest regression for classification, achieving an accuracy of over 94% in differentiating childhood from puberty/adolescence, slightly higher than our BLSTM model (86%, Fig. 1A). Notably, Vandenbosch et al. utilized a considerably larger EEG database consisting of 852 children and 1816 adolescents, using them iteratively as training and testing samples. Upon closer examination of Vandenbosch’s study, the classification accuracy in the children group was reported as 86.5% (736/851), which was lower than our own data (24/25, 96% accuracy). Despite having a substantially smaller EEG sample size, we were able to develop a cognitive age classifier similar to the previous study. However, when we attempted to further classify EEGs into young children (0–5 years), older children (6–11 years), and adolescents (12–20 years), the overall accuracy dropped to only 69.3%. This decrease in accuracy can likely be attributed to the notable individual variation in cognitive abilities. Even among typically developing children, their intelligence quotients (IQs), as quantified by standardized intelligence tests like the Wechsler Intelligence Scale for Children-V, span a range from 70 to 130. Consequently, it is not surprising that children of consecutive ages may exhibit similar cognitive abilities, leading to modest accuracy when classifying cognitive age based on EEG data. To demonstrate that the BLSTM model can capture cognitive ability, we expanded our analysis to include EEGs from patients with neurodevelopmental disorders. Through multivariate analysis, we found that intellectual disability (ID) emerged as the primary factor influencing classification accuracy. This finding supports the notion that the BLSTM model effectively differentiates EEG patterns associated with individuals’ cognitive abilities.

Early recognition of children with developmental delays and ID is essential for their entry in to comprehensive intervention services to help improve their cognitive and social outcomes^35^. Parental observation alone is often inadequate for identifying developmental delays, and the development of screening tests and surveillance can help in the early detection of at-risk children^36,37^. In addition to traditional developmental tests, EEGs an reflect neurological disease activity or serve as neurophysiological markers for various neurodevelopmental disorders^38^. However, bulky EEG instruments are uncomfortable and inconvenient for users to perform daily life tasks^39^, and EEG interpretation is a time-consuming specialized skill, which are barriers to clinical utility. However, AI-supported EEG interpretations are increasingly feasible with advances in automated learning programs. We have illustrated that LSTM with clinical EEG data can facilitate cognitive age determination. Moreover, mobile EEG technologies have been developed to overcome instrumental limitations and to offer a solution for real-time neurophysiological monitoring and intervention^40^. For example, attention deficit/hyperactivity disorder (ADHD)-affected children may struggle to stay focused for extended periods, and concomitant quantitative EEG monitoring of their attention-to-resting alpha power ratio would be a good indicator of attention ability, which could benefit in individualized intervention services^38^. However, this was a start-up study, so further hardware and software improvements are key to the application of EEGs for neurodevelopmental disease recognition and intervention.

With the advancements in parallel computing technology, the Transformer model has been introduced to enhance the computational efficiency of recurrent patterns^41^. Unlike traditional recurrent models such as BLSTMs, which process computations sequentially or in reverse chronological order^42^, the Transformer model computes a correlation matrix between the current input and other time vectors. This matrix is then utilized to apply a weighting function that highlights important parts of the sequence relevant to the given task^26^. Additionally, all computations in Transformer models can be executed in parallel on user devices, reducing training and testing time^43^. Therefore, efficient parallelization of computations is crucial when implementing Transformer systems. To evaluate the efficiency of the Transformer and BLSTM models, we conducted experiments under three different conditions, measuring the average inference time. The physical CPU and GPU used were “Intel(R) Core(TM) i7-10,700” and “NVIDIA RTX A2000,” respectively. To generate the results, we first calculated the time required to predict target clusters for each input EEG, and then averaged these results to obtain the average processing time. Consequently, the average processing time for BLSTM was 0.10 s, while for the Transformer, it was 0.14 s. The Transformer system exhibited the longest computation time. This is attributed to the Transformer model containing approximately 15 million parameters and flattened functions, necessitating significant hardware resources and resulting in high computational costs. However, by adjusting the (head size, element size) to (128, 8) for multiple components, the average processing time of the Transformer model was reduced to 0.12 s. Nevertheless, it still remained higher than that of BLSTM. Additionally, the adjusted Transformer model had around 500,000 parameters. Interestingly, the age classification accuracy of the adjusted Transformer model decreased significantly to 38.67%, lower than that of the original Transformer setting. These results indicate that the Transformer model encounters difficulties in effectively handling temporally distinct EEG signals for an age classification task.

This study has some limitations. First, it lacked the complete psychometric profiles of EEGs from neurotypical patients; however, each patient in this study was interviewed by an experienced neurologist, psychologist, or physiatrist, and patients with marginal intelligence were excluded from the study. Second, we collected EEGs from a clinical EEG laboratory where patients had been referred for an EEG study under certain clinical diagnoses, which might be a potential bias. Third, only 300 EEGs from neurotypical patients aged between 8 months and 20 years were included in the LSTM training; therefore, we could not divide the cognitive age range into more groups. Furthermore, all EEG samples were collected in one single institution in Taiwan which possibly influence the generalizability of this model. Additionally, the limited dataset size does pose a risk of overfitting. Nonetheless, it’s worth mentioning that the sample size in our study is comparable to previous machine-learning-based models for age prediction from EEG signals^44–46^. By incorporating data from different geographic regions and institutions, we can enhance the generalizability and robustness of the model. Another limitation of the BLSTM and Transformer models employed in this study is their inability to pinpoint specific brain ages; they can only differentiate EEG samples into three broad age groups. In contrast, regression models like the RVM and RF model, as utilized in Vandenbosch et al.’s study^15^, have demonstrated the capability to estimate an individual EEG sample’s age, achieving an accuracy of 1.22 years in that particular study. In the future, a promising approach could involve combining two or more AI models to effectively ascertain brain age from EEG data. This integration may lead to the development of a practical instrument for assessing developmental status. Despite these limitations, our study has proved the concept that EEG can represent an individual’s cognitive abilities, and deep learning algorithms, including BLSTM, can be applied to automatic EEG interpretation.

## Conclusion

In this study, we generated a cognitive age classifier that can potentially be applied to clinical services using clinical EEG data and an LSTM deep learning algorithm. This classifier proved the concept that EEG data can serve as a cognitive ability indicator in children and adolescents and can be used as a neurological biomarker for neurodevelopmental disorders, particularly ID. However, the classifier was limited by only dividing cognitive age into young children, older children, and adolescents, with a classification accuracy of 69.3%. In the future, we plan to incorporate a larger and multi-institutional EEG dataset comprising children and adolescents, aiming to enhance the generalization capabilities of the BLSTM model.

### Supplementary Information

Supplementary Figure 1.

## Data Availability

The raw data supporting the conclusions of this article will be made available by the authors without undue reservation.

## References

[1] Swaiman KF, Ashwal S, Ferriero DM, Schor NF (2018). Swaiman’s Pediatric Neurology: Principles and Practice.

[2] Parenti I, Rabaneda LG, Schoen H, Novarino G (2020). Neurodevelopmental disorders: From genetics to functional pathways. Trends Neurosci..

[3] Patel DR, Cabral MD, Ho A, Merrick J (2020). A clinical primer on intellectual disability. Transl. Pediatr..

[4] Anatürk M (2021). Prediction of brain age and cognitive age: Quantifying brain and cognitive maintenance in aging. Hum. Brain Mapp..

[5] Shokri-Kojori E, Bennett IJ, Tomeldan ZA, Krawczyk DC, Rypma B (2021). Estimates of brain age for gray matter and white matter in younger and older adults: Insights into human intelligence. Brain Res..

[6] Murias K, Moir A, Myers KA, Liu I, Wei XC (2017). Systematic review of MRI findings in children with developmental delay or cognitive impairment. Brain Dev..

[7] Al Sawaf, A., Gudlavalleti, A. & Murr, N. *EEG basal cortical rhythms. StatPearls*. Treasure Island (FL): StatPearls publishing. *Copyright* © 2021. (StatPearls Publishing LLC, 2021). 30422580

[8] Kaminska A, Eisermann M, Plouin P (2019). Child EEG (and maturation). Handb. Clin. Neurol..

[9] Bazanova OM, Vernon D (2014). Interpreting EEG alpha activity. Neurosci. Biobehav. Rev..

[10] Castro Conde JR (2020). Assessment of neonatal EEG background and neurodevelopment in full-term small for their gestational age infants. Pediatr. Res..

[11] De Ridder J (2020). Prediction of neurodevelopment in infants with tuberous sclerosis complex using early EEG characteristics. Front. Neurol..

[12] Sun H (2019). Brain age from the electroencephalogram of sleep. Neurobiol. Aging.

[13] Paixao L (2020). Excess brain age in the sleep electroencephalogram predicts reduced life expectancy. Neurobiol. Aging.

[14] Dimitriadis SI, Salis CI (2017). Mining time-resolved functional brain graphs to an EEG-based chronnectomic brain aged index (CBAI). Front. Hum. Neurosci..

[15] Vandenbosch M, van’t Ent D, Boomsma DI, Anokhin AP, Smit DJA (2019). EEG-based age-prediction models as stable and heritable indicators of brain maturational level in children and adolescents. Hum. Brain Mapp..

[16] Kaushik P, Gupta A, Roy PP, Dogra DP (2018). EEG-based age and gender prediction using deep BLSTM-LSTM network model. IEEE Sens. J..

[17] Jusseaume K, Valova I (2022). Brain age prediction/classification through recurrent deep learning with electroencephalogram recordings of seizure subjects. Sensors.

[18] Medvedev AV, Agoureeva GI, Murro AM (2019). A long short-term memory neural network for the detection of epileptiform spikes and high frequency oscillations. Sci. Rep..

[19] Kim Y, Choi A (2020). EEG-based emotion classification using long short-term memory network with attention mechanism. Sensors (Basel).

[20] Phutela, N., Relan, D., Gabrani, G., Kumaraguru, P. & Samuel, M. Stress classification using brain signals based on LSTM network. *Comput. Intell. Neurosci. *7607592 (2022). 10.1155/2022/7607592 PMC9071939 35528348

[21] Sharma SD, Sharma S, Singh R, Gehlot A, Priyadarshi N, Twala B (2022). Stress detection system for working pregnant women using an improved deep recurrent neural network. Electronics..

[22] Maitin AM, Romero Muñoz JP, García-Tejedor ÁJ (2022). Survey of machine learning techniques in the analysis of EEG signals for Parkinson’s disease: A systematic review. Appl. Sci. (Basel).

[23] Kumar S, Sharma A, Tsunoda T (2019). Brain wave classification using long short-term memory network based OPTICAL predictor. Sci. Rep..

[24] Xu G, Ren T, Chen Y, Che W (2020). A one-dimensional CNN-LSTM model for epileptic seizure recognition using EEG signal analysis. Front. Neurosci..

[25] Sakalle A, Tomar P, Bhardwaj H, Acharya D, Bhardwaj A (2021). A LSTM based deep learning network for recognizing emotions using wireless brainwave driven system. Expert Syst. Appl..

[26] Vaswani, A. *et al.* Attention is all you need. *Adv. Neural Inf. Process Syst.* **30** (2017).

[27] He S, Grant PE, Ou Y (2021). Global-local transformer for brain age estimation. IEEE Trans. Med. Imag..

[28] Cai H, Gao Y, Liu M (2023). Graph transformer geometric learning of brain networks using multimodal MR images for brain age estimation. IEEE Trans Med Imaging..

[29] Hochreiter S, Schmidhuber J (1997). Long short-term memory. Neural Comput..

[30] Zen, H. & Sak, H. editors. Unidirectional long short-term memory recurrent neural network with recurrent output layer for low-latency speech synthesis. In *2015 IEEE International Conference on Acoustics, Speech and Signal Processing (ICASSP)* (IEEE, 2015).

[31] Graves A, Schmidhuber J (2005). Framewise phoneme classification with bidirectional LSTM and other neural network architectures. Neural Netw..

[32] Gers FA, Schmidhuber J, Cummins F (2000). Learning to forget: Continual prediction with LSTM. Neural Comput..

[33] Bengio Y, Lee H (2015). Editorial introduction to the neural networks special issue on deep learning of representations. Neural Netw..

[34] Hinton GE, Salakhutdinov RR (2006). Reducing the dimensionality of data with neural networks. Science.

[35] Guralnick MJ (2017). Early intervention for children with intellectual disabilities: An update. J. Appl. Res. Intellect. Disabil..

[36] Vitrikas K, Savard D, Bucaj M (2017). Developmental delay: When and how to screen. Am. Fam. Phys..

[37] Kim S (2022). Worldwide national intervention of developmental screening programs in infant and early childhood. Clin. Exp. Pediatr..

[38] Tsai LP, Wang SS, Chee SY, Wong SB (2022). Dynamic changes in the quantitative electroencephalographic spectrum during attention tasks in patients with Prader–Willi syndrome. Front. Genet..

[39] Chen, X. & Wang, Z. J. editors. Design and implementation of a wearable, wireless EEG recording system. In *2011 5th International Conference on Bioinformatics and Biomedical Engineering* (IEEE, 2011).

[40] Lau-Zhu A, Lau MPH, McLoughlin G (2019). Mobile EEG in research on neurodevelopmental disorders: Opportunities and challenges. Dev. Cogn. Neurosci..

[41] Lee, Y.-E. & Lee, S.-H. editors. EEG-transformer: Self-attention from transformer architecture for decoding EEG of imagined speech. in *2022 10th International Winter Conference on Brain-Computer Interface (BCI)* (IEEE, 2022).

[42] Gao, J., Zhang, H., Lu, P., & Wang, Z. An effective LSTM recurrent network to detect arrhythmia on imbalanced ECG dataset. *J Healthc. Eng.* **2019** (2019). 10.1155/2019/6320651 PMC6815557 31737240

[43] Xiong, Y., Du, B. & Yan, P., editors. Reinforced transformer for medical image captioning. *Machine Learning in Medical Imaging: 10th International Workshop, MLMI 2019, Held in Conjunction with MICCAI 2019, Shenzhen, China, October 13, 2019, Proceedings 10* (Springer, 2019).

[44] Paiva LRMd, Pereira AA, Almeida MFSd, Cavalheiro GL, Milagre ST, Andrade AdO (2012). Analysis of the relationship between EEG signal and aging through Linear Discriminant Analysis (LDA). Rev. Brasil. Eng. Biomed..

[45] Nguyen, P., Tran, D., Vo, T., Huang, X., Ma, W. & Phung, D., editors. EEG-based age and gender recognition using tensor decomposition and speech features. In *Neural Information Processing: 20th International Conference, ICONIP 2013, Daegu, Korea, November 3–7, 2013 Proceedings, Part II 20* (Springer, 2013).

[46] Kaur B, Singh D, Roy PP (2019). Age and gender classification using brain–computer interface. Neural Comput. Appl..

