# Author Correction: Application of bi-directional long-short-term memory network in cognitive age prediction based on EEG signals

**DOI:** 10.1038/s41598-024-53922-3

**Published:** 2024-03-05

**Authors:** Shi‑Bing Wong, Yu Tsao, Wen‑Hsin Tsai, Tzong‑Shi Wang, Hsin‑Chi Wu, Syu‑Siang Wang

**Affiliations:** 1 Department of Pediatrics, Taipei Tzu Chi Hospital, Buddhist Tzu Chi Medical Foundation, New Taipei City, Taiwan; 2 School of Medicine, Tzu Chi University, Hualien, Taiwan; 3 Research Center for Information Technology Innovation, Academia Sinica, Taipei, Taiwan; 4 Department of Psychiatry, Taipei Tzu Chi Hospital, Buddhist Tzu Chi Medical Foundation, New Taipei City, Taiwan; 5 Department of Physical Medicine and Rehabilitation, Taipei Tzu Chi Hospital, Buddhist Tzu Chi Medical Foundation, New Taipei City, Taiwan; 6 Department of Electrical Engineering, Yuan Ze University, Taoyuan, Taiwan

Correction to: *Scientific Reports* 10.1038/s41598-023-47606-7, published online 18 November 2023

In the original version of this Article a part of the title was erroneously omitted.

“Application of bidirectional long-short-term memory network in cognitive age prediction”

now reads:

“Application of bi-directional long-short-term memory network in cognitive age prediction based on EEG signals”

The original Article has been corrected.

