# Supplementary material for: Application of bi-directional long-short-term memory network in cognitive age prediction based on EEG signals
Source: Sci Rep. 2023 Nov 18;13:20197. doi: 10.1038/s41598-023-47606-7 (PMC10657465; doi:10.1038/s41598-023-47606-7)
Supplement: Supplementary file 1 — Supplementary Figure 1. [file 41598_2023_47606_MOESM1_ESM.docx]

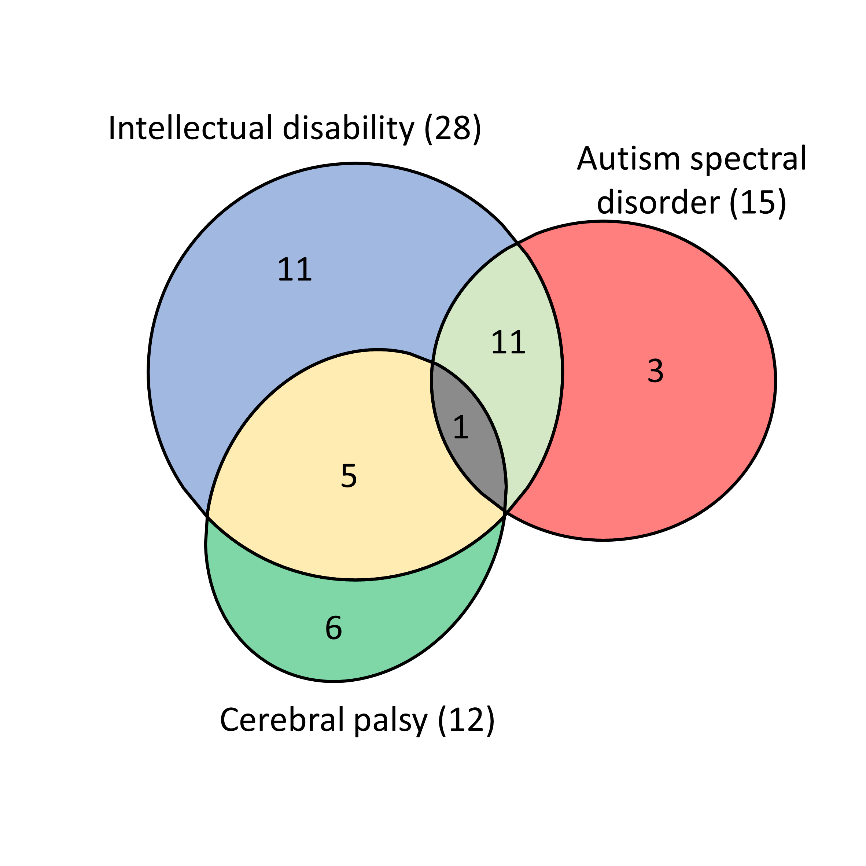


Supplementary figure 1. Overlapping pie chart representing the distribution of patients with intellectual disability, autism spectrum disorder, and cerebral palsy.
